# Anisotropic characteristics and improved magnetic performance of Ca–La–Co-substituted strontium hexaferrite nanomagnets

**DOI:** 10.1038/s41598-020-72608-0

**Published:** 2020-09-28

**Authors:** Jimin Lee, Eun Jae Lee, Tae-Yeon Hwang, Jongryoul Kim, Yong-Ho Choa

**Affiliations:** 1 Department of Materials Science and Chemical Engineering, Hanyang University, 55, Hanyangdaehak-ro, Sangnok-gu, Ansan-si, 15588 Gyeonggi-do Korea; 2 Center for Quantum Information, Korea Institute of Science and Technology (KIST), 5, Hwarang-ro 14-gil, Seongbuk-gu, Seoul, 02792 Korea

**Keywords:** Materials science, Nanoscience and technology, Physics

## Abstract

Recent studies on next-generation permanent magnets have focused on filling in the gap between rare-earth magnets and rare-earth-free magnets, taking into account both the cost-effectiveness and magnetic performance of the magnetic materials. As an improved rare-earth-free magnet candidate, here, Ca-substituted M-type Sr-lean hexaferrite particles within a nano- to micro-scale regime, produced using an ultrasonic spray pyrolysis method, are investigated. Theoretically, the maximum coercivity (*H*_c_) can be achieved in submicron Sr-ferrite crystals (i.e., 0.89 μm). The plate-like resultants showed a significant enhancement in *H*_c_, up to a record high of 7880.4 Oe, with no deterioration in magnetization (*M*: 71–72 emu/g). This resulted in more favorable magnetic properties than those of the traditional Sr–La–Co ferrites. On the basis of microstructural analysis and fitting results based on the law of approach to saturation method, the Ca-substitution effects on the change in size and anisotropic characteristics of the ferrite particles, including pronounced lateral crystal growth and a strong increase in magnetocrystalline anisotropy, are clearly demonstrated. The cost-effective, submicron, and Ca-substituted Sr-ferrite is an excellent potential magnet and moreover may overcome the limitations of traditional hard magnetic materials.

## Introduction

As the world pursues higher energy efficiency for miniaturized devices such as small motors in hybrid electric vehicles, the demand for ultra-high-performance permanent magnets is rapidly increasing. There are two types of permanent magnets: rare-earth (RE) magnets (e.g., SmCo and NdFeB) with broadly outstanding magnetic characteristics but they have high concentrations scarce RE elements; and the diametrically opposed RE-free magnets (e.g., Sr-ferrite and Ba-ferrite), which possess relatively poor magnetic properties, but are less expensive and have excellent oxidation resistance^1^. So-called “*gap magnets*” have been introduced as a compromise to fill the gap between RE magnets and RE-lean magnets, given both their lower material costs and good magnetic performance^2,3^.

To date, many reports have suggested M-type Sr-hexaferrite (SrFe_12_O_19_) with elemental substitutions as a viable candidate for a high-performance magnet, by the substitution of RE ions and other cations such as Sm^3+^^4^, Sm^3+^–Co^2+^^5^, La^3+^–Sm^3+^^6^, La^3+^–Co^2+^^7,8^, Nd^3+^^9^, Nd^3+^–Y^3+^^10^, and Nd^3+^–Co^2+^^11^. Among these gap magnets, partial La^3+^–Co^2+^-substituted Sr-ferrite has received great attention due to the successful enhancement in its intrinsic coercivity (*H*_c_)^8,12^. Interestingly, upon non-RE substitution, Al^3+^ for Fe^3+^ in SrFe_12_O_19_, the *H*_c_ increased up to tens of kOe, although this came at the expense of the saturation magnetization (*M*_s_), which dropped from ~ 60 emu/g to ~ 10 emu/g. This led to a deterioration in the maximum energy product ((*BH*)_max_), which is the most important magnetic parameter^13,14^.

In this regard, elemental substitution should enhance *H*_c_ without sacrificing the *M* value. We have found that Sr-ferrites with earth-abundant Ca-substitution need further research in terms of their microstructure and magnetic properties. There are a few previous reports on M-type hexaferrites with Ca-substitution using conventional solid-state reaction routes^15–20^; however, the solid-state process does not allow adequate control of the particle size, morphology, or homogeneity, resulting in ambiguities in the relationship between the effect of elemental substitution and the microstructural properties^21^. Some reports developed an enhancement in *H*_c_ upon Ca-substitution; however, the reason has not been fully corroborated. From microstructural, magnetic, and anisotropic points-of-view, we have found that the Ca-substitution effect is still in need of further research.

The goal of this study is to synthesize Ca-substituted Sr-ferrites possessing enhanced *H*_c_ without *M*_s_ deterioration and to elucidate the Ca-substitution effects on the morphological, crystallographic, and magnetic performance of the ferrite particles. Motivated by the past success of La–Co-substitution in Sr-ferrite, we concurrently searched for the optimal amount of La–Co-substitution. We used salt-assisted ultrasonic spray pyrolysis (SA-USP) to prepare Ca-substituted, M-type Sr-hexaferrites, Sr_0.75-*x*_La_0.25_Ca_*x*_Fe_11.8_Co_0.2_O_19_ (Ca content *x* = 0.00, 0.05, 0.10, 0.15, 0.20, 0.25, 0.30, 0.40, and 0.60) in a submicron regime. Recently, the application of spray pyrolysis in a salt matrix for the synthesis of single crystals with exquisitely controlled homogeneity has become an attractive research area^22^.

## Methods

### Chemicals

To synthesize a Ca–La–Co-substituted SrFe_12_O_19_ submicron powder via SA-USP, we used the following raw materials without further purification: strontium (II) nitrate (Sr(NO_3_)_2_, 99.0%; Sigma-Aldrich, USA), lanthanum (III) nitrate hexahydrate (La(NO_3_)_3_·6H_2_O, 99.0% up; Sigma-Aldrich, USA), calcium (II) nitrate tetrahydrate (Ca(NO_3_)_2_·4H_2_O, 99.9%; Merck, Germany), iron (III) nitrate nonahydrate (Fe(NO_3_)_3_·9H_2_O, 98%; Junsei Chemical Co., Ltd., Japan), and cobalt (II) nitrate hydrous (Co(NO_3_)_2_·6H_2_O, 99.9% up; Kojundo Chemical Laboratory Co., Ltd., Japan) as metal precursors, and sodium chloride (NaCl, 99.0%; Daejung Chemical & Metals Co., Ltd., South Korea) as a salt matrix.

### Hexaferrite particle synthesis through the SA-USP process

To prepare the precursor solution for Sr-ferrite with various degrees of Ca-substitution, a series of stoichiometric amounts of nitrate sources were dissolved in 300 mL of distilled water according to the formula Sr_0.75−*x*_La_0.25_Ca_*x*_Fe_11.8_Co_0.2_O_19_ (*x* = 0.00, 0.05, 0.10, 0.15, 0.20, 0.25, 0.30, 0.40 and 0.60), to give 65 mmol of the all amounts of the cations (i.e., Sr^2+^, La^3+^, Ca^2+^, Fe^3+^, and Co^2+^). All precursor solutions contained a fixed concentration of NaCl (0.92 M) to regulate the reaction conditions.

For the USP process, the precursor solution, which was homogeneously stirred for 3 h, was fed into a cylindrical quartz tube with side arms (see the schematic of a laboratory-scale USP setup in Fig. S1a). The precursor solution was first atomized by an ultrasonic mist generator (1.7 MHz of frequency), and the atomized droplets were introduced into a tube furnace by an O_2_ carrier gas (flow rate of 2 L/min), and then thermally pyrolyzed through the heating stage in O_2_ currents at 650 °C. A subsequent calcination process of the trapped intermediate particles was performed at 1050 °C (in air, for 1 h) to complete the SrFe_12_O_19_ phase formation^23^. The calcined samples were rinsed with distilled water to remove most of the residual NaCl, and were dried overnight in a vacuum oven. (See the phase and morphology of the synthesized particles in Fig. S1b and c, respectively.) The overall procedure was slightly modified from a previous method that we describe in detail elsewhere^7,22^.

### Characterization

Morphological characterization and particle size measurement of the Ca–La–Co-substituted SrFe_12_O_19_ nanoparticles were performed by using field emission scanning electron microscopy (FE-SEM; MIRA-3, Tescan, Czech Republic). X-ray diffractometry (XRD; D/MAX-2500/PC, Rigaku Co., Japan) was employed for a crystal-structural characterization of the magnetic powder. The Rietveld refinements on the XRD patterns were performed by using the JAVA-based refinement program (Materials Analysis Using Diffraction; MAUD). The magnetic performance of the Sr-ferrite powder at room temperature were examined by vibrating sample magnetometry (VSM; VSM7410, Lake Shore Cryotronics, Inc., USA). There was no additional magnetic alignment and sintering processes.

## Results and discussion

### Morphology of the ferrites with different Ca contents

**Figure 1:**
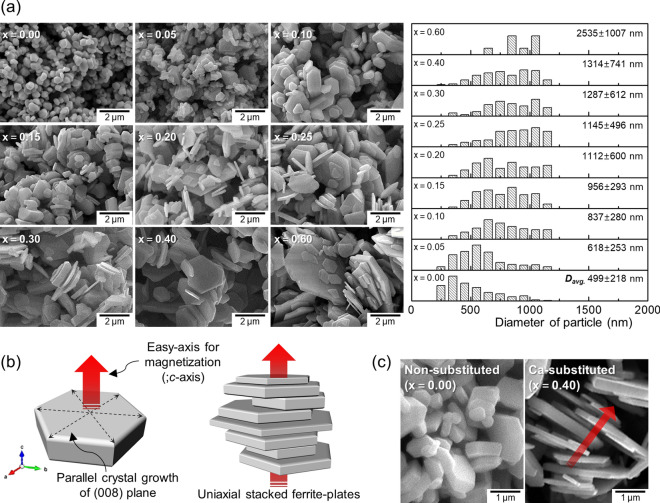
(**a**) FE-SEM micrographs and particle diameter distribution histograms of the samples with different Ca contents (*x*) in Sr_0.75−*x*_La_0.25_Ca_*x*_Fe_11.8_Co_0.2_O_19_ (0.00 ≤ *x* ≤ 0.60); (**b**) schematic of the hexagonal Sr-ferrite plates with preferred orientation; (**c**) FE-SEM images of (left) the Sr-ferrite particles with no Ca addition, and (right) partial Ca^2+^-substituted Sr-ferrite powder with a higher aspect ratio.

FE-SEM micrographs of the series of Ca–La–Co-substituted ferrite samples and their size distributions are depicted in Fig. 1a as a function of Ca content (*x*). Table 1 contains the numerical data, including the mean particle sizes with corresponding standard deviations. As the amount of Ca increased up to 0.40, there was a noticeable change in not only the thickness, but also in the diameter of the Sr-ferrite particles (ranging from the submicron to micro scale), while they became more plate-like in shape with a high aspect ratio, up to 9.31. Thus, it can be inferred that the Ca-substitution affected the anisotropic parameters, inducing the predominantly lateral crystal growth in the hexagonal ferrites^24^. Even though there was salt (i.e., NaCl) introduced to prepare the resultant particles with precisely controlled homogeneity of the dimension, additional Ca-substitution (*x* > 0.40) led to abnormal grain growth, resulting in a broad particle diameter distribution that mainly reflected Ostwald ripening^25^. Figure 1b presents a schematic of the evolving hexagonal crystal structures of the Sr-ferrites, with their preferred orientation of particle-stacking. Even though there was no applied external magnetic field, the pronounced crystal growth perpendicular to the *c*-axis with a high aspect ratio (i.e., the <00*l*> direction) coming from the Ca-substitution led to the easy stacking of the plate-like ferrite particles along the *c*-axis, in good agreement with the experimental data (Fig. 1c).

**Table 1:** Mean particle sizes, standard deviations, and aspect ratios of the M-type Sr_0.75−*x*_La_0.25_Ca_*x*_Fe_11.8_Co_0.2_O_19_ ferrite samples as a function of Ca content (*x*).

Ca^2+^ content (*x*)	Diameter (*D*)		Thickness (*t*)		Aspect ratio (*D*/*t*)
	Average (nm)	Std. Dev (nm)	Average (nm)	Std. Dev (nm)	
0.00	499.2	217.7	186.3	62.2	2.7
0.05	618.2	252.9	191.4	58.8	3.2
0.10	837.2	280.5	210.6	51.3	4.0
0.15	955.5	293.0	174.0	48.0	5.5
0.20	1111.5	599.6	151.6	46.3	7.3
0.25	1145.0	495.6	160.4	62.4	7.1
0.30	1286.8	611.9	182.4	62.2	7.1
0.40	1314.3	740.9	141.2	62.5	9.3
0.60	2535.6	1007.3	299.3	100.7	8.5

### Crystallographic characteristics upon Ca-substitution

**Figure 2:**
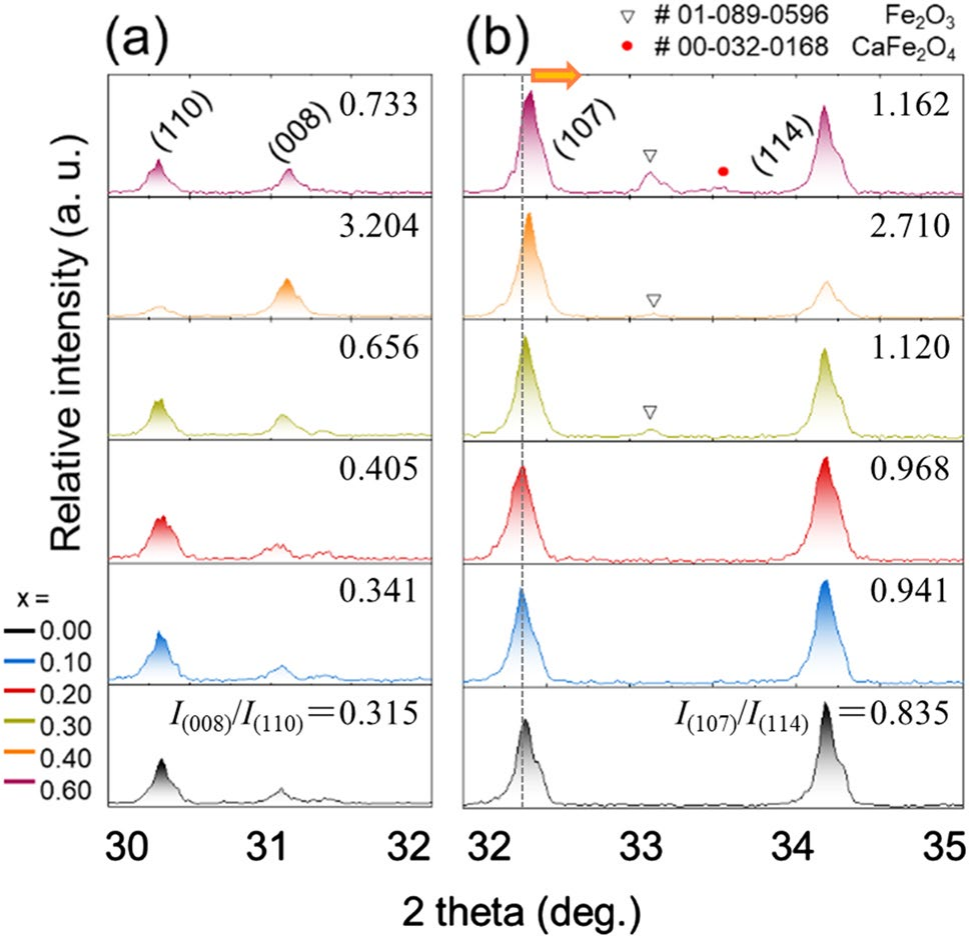
Enlarged view of the most intense XRD patterns of the Ca-substituted Sr-hexaferrite powder with various degrees of Ca-substitution (*x*) in the 2θ range of (**a**) 30°–32°, and (**b**) 32°–35°, respectively. The peak position shifts toward a higher angle as the amount of Ca^2+^-substitution increases.

Figure 2 shows the normalized X-ray diffraction patterns of the series Sr_0.75−*x*_La_0.25_Ca_*x*_Fe_11.8_Co_0.2_O_19_ (*x* = 0.00, 0.05, 0.10, 0.15, 0.20, 0.25, 0.30, 0.40 and 0.60) (see their entire XRD patterns in the 2θ range of 20–80° in Fig. S2). For the samples with Ca content (*x*) ranging from 0.00 ≤ *x* ≤ 0.20, the diffraction pattern indicated a pure hexagonal SrFe_12_O_19_ phase listed in the JCPDS card, No. 80-1197, suggesting that the Ca^2+^ ions were all incorporated into the lattice of the Sr-hexaferrite^16^. At *x* ≥ 0.30, the pattern was clearly composed of the crystalline SrFe_12_O_19_ as well as small amounts of foreign phases, particularly Fe_2_O_3_ (No. 89-0596) and CaFe_2_O_4_ (No.32-0168). In this regard, Rietveld refinements for the Sr-hexaferrite particles with different Ca contents were conducted and the quantitative results are shown in Fig. S3. The introduction of secondary phases with increasing Ca concentration can be described by the following reactions (Eqs. 1 and 2). For the sake of demystifying the equations, La–Co substitutions are neglected^26^:

1 $$aSr\left( {NO_{3} } \right)_{2} + 2bFe\left( {NO_{3} } \right)_{3} + cCa\left( {NO_{3} } \right)_{2} \to aSrO + bFe_{2} O_{3} + cCaO$$

2 $$aSrO + bFe_{2} O_{3} + cCaO \to pSr_{1 - x} Ca_{x} Fe_{12} O_{19} + qCaFe_{2} O_{4} + rFe_{2} O_{3}$$

where *a*, *b*, *c*, *p*, *q*, and *r* are constants. Based on the reaction equations and experimental data, we deduced that the maximum amount of Sr^2+^ substituted by Ca^2+^ can reach *x* < 0.30, while surplus Ca^2+^ cations are prone to induce the formation of spinel Ca-ferrite (CaFe_2_O_4_) rather than substitute the Sr^2+^ site in the ferrites. The *x* < 0.30 threshold might also be related to the formation of solid solution in the CaO–SrO binary system during thermal pyrolysis (Eq. 1), due to the similar crystal structures, ionic radii, and valences of the two oxides^27,28^. Besides, this pseudo-binary-phase system has different thermodynamic behavior compared to the single-phase counterparts, thus it is able to induce a difference in the degree of crystal growth during the calcination process. Based on this, Ca-substitution seems to have the effect of accelerating ferrite particle growth to be more platelet-like. When the maximum Ca concentrations were introduced in Sr_0.75−*x*_La_0.25_Ca_*x*_Fe_11.8_Co_0.2_O_19_, i.e., there is no Sr source (*x* = 0.75), CaFe_2_O_4_ and Fe_2_O_3_ X-ray patterns are dominantly observed, while the hexaferrite peaks are relatively weak; this implies that high Ca contents (*x* > 0.60) weaken the formation of hexaferrite. However, we obtained interesting results from the FE-SEM micrograph showing micro-scale hexagonal plates (composing of a hexaferrite phase) with diameters ranging from 10 to 50 μm, which exceeds the diameter of the ferrites with *x* = 0.60 (2.5 μm). That is, the Sr-free hexagonal microplates clearly back up the Ca substitution effect on the growth of particles to be more plate-like in shape, with improved crystallinity (see the data in Fig. S4).

The shift in the SrFe_12_O_19_ peak position toward a higher angle, which results from a considerable change in the lattice parameters (Figure S5), was observed as the Ca content in Sr_0.75−*x*_La_0.25_Ca_*x*_Fe_11.8_Co_0.2_O_19_ continuously increased. This could be mainly attributed to the fact that the ionic radius of the Ca^2+^ ion (0.099 nm) was smaller than that of the Sr^2+^ ion (0.110 nm), leading to lattice shrinkage during Sr-ferrite phase formation^29^. Interestingly, the relative intensity ratio of reflections (008) to (110) (= *I*_(008)_/*I*_(110)_) clearly increased with increasing *x* from 0.00 to 0.40 (Fig. 2a). It can be inferred that the plate-like Ca-substituted hexaferrites, originating from dominant crystal growth perpendicular to their *c*-axis, are able to partially and spontaneously orient themselves uniaxially (<00*l*>), thereby leading to the change in relative intensity ratio of the reflections without an external magnetic field^30^. When the Ca content increased up to 0.60, the plate-like microparticles were aligned in a haphazard manner, similar to the non-substituted ferrite nanoparticles, due to their broad size distribution and the incorporated byproduct (i.e., Fe_2_O_3_ and CaFe_2_O_4_). Likewise, the behavior of *I*_(107)_/*I*_(114)_ can be understood in the same way (Fig. 2b).

### Magnetic performance as a function of Ca content

**Figure 3:**
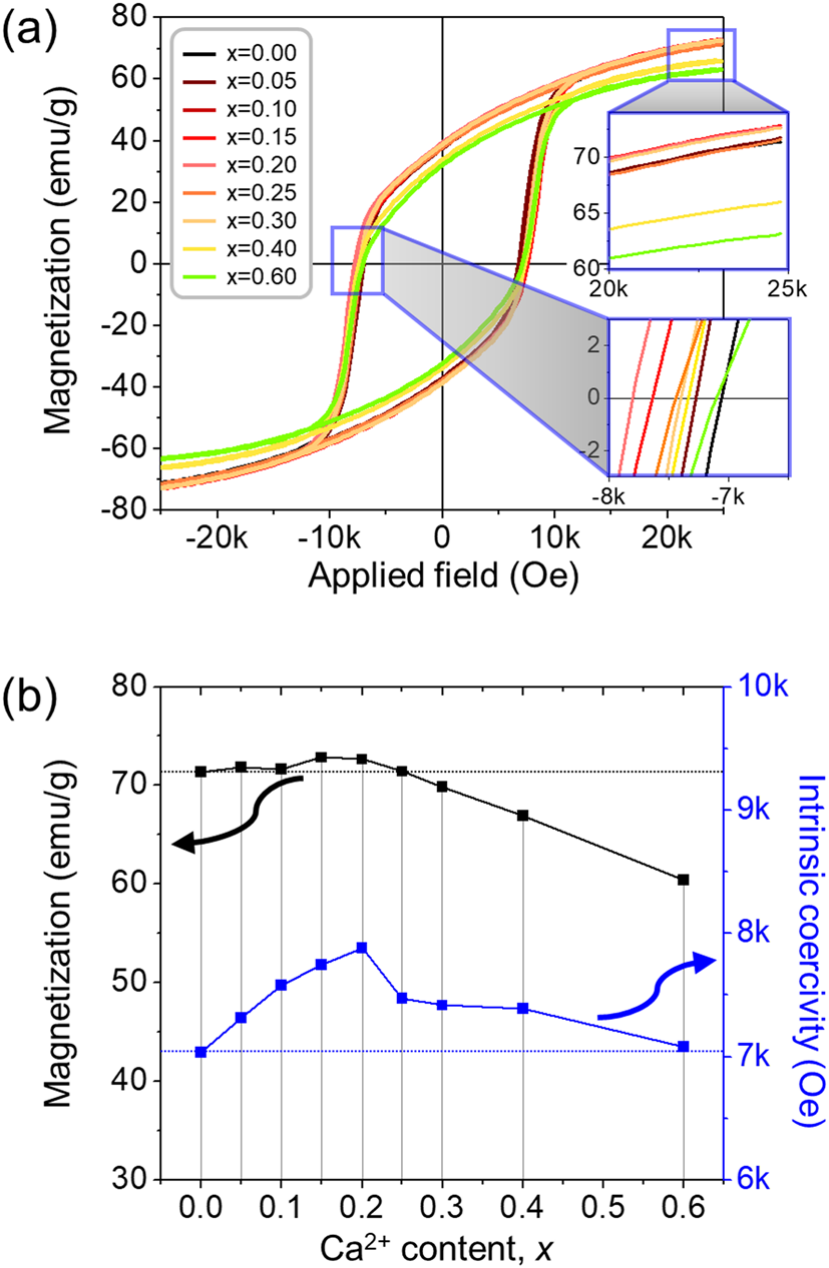
(**a**) *M*–*H* curves, and (**b**) variations of *M*_25kOe_ and *H*_c_ of Sr_0.75−*x*_La_0.25_Ca_*x*_Fe_11.8_Co_0.2_O_19_ hexaferrite (0.00 ≤ *x* ≤ 0.60) with respect to Ca content (*x*), obtained under the applied field of 25 kOe at room temperature. The enlarged views in (**a**) clearly show the *M*–*H* loops.

Magnetic measurements of the Sr-hexaferrite particles with different Ca contents were conducted at room temperature (Fig. 3a). Regardless of the quantity of Ca-substitution in the Sr-ferrite, all hysteresis loops showed single-phased ferromagnetic behavior without kinks, even though a small amount of the antiferromagnetic Fe_2_O_3_ phase was incorporated in the samples with *x* ≥ 0.30^31^. Fig. 3b illustrates the dependence of the maximum magnetization at 25 kOe (*M*_25kOe_) and the intrinsic coercivity (*H*_c_) of each hexaferrite sample on the amount of Ca. Table 2 provides the numerical data, including *M*_25kOe_, remanence (*M*_r_), *H*_c_, and squareness. Clearly, from *x* = 0.00 to 0.30, the *M*_25kOe_ values did not degrade, whereas the maximum value of *H*_c_ peaked at (~ 7880.4 Oe) at *x* = 0.20 and then decreased, but remained above the value of the non-substituted ferrites. Generally speaking, the inherent magnetic parameter *M* can fall from the theoretical value (e.g., ~ 72 emu/g for pristine Sr-ferrites^32^) mainly due to a decrease in either phase purity or in the crystallinity of the magnetic particles. The saturation magnetization (*M*_s_) of nano-scaled Fe_2_O_3_ as reported in a previous study is only ~ 10 emu/g^33^. Furthermore, the extrinsic factor, *H*_c_, can vary according to a complex set of variables such as the grain size, particle shape, degree of particle orientation, and the particle density^19,34^; due to the increase of the demagnetization factor, *H*_c_ can decrease a fair amount when the magnetic particles become more plate-like^18^. Accordingly, with cationic substitution, the value of *M* continued to deteriorate from the theoretical *M*, even when *H*_c_ was maintained or slightly increased to a value more than that of the pristine powder, as has been well documented in many previous studies^11,13–16^.

**Table 2:** Effect of Ca-substitutions on the magnetic properties of a maximum magnetization at 25 kOe (*M*_25kOe_), remanence (*M*_r_), intrinsic coercivity (*H*_c_), squareness (*M*_r_/*M*_25kOe_), and calculated maximum energy product ((*BH*)_max_) for the M-type Sr_0.75−*x*_La_0.25_Ca_*x*_Fe_11.8_Co_0.2_O_19_ ferrite samples.

Ca^2+^ content (*x*)	*M*_25kOe_ (emu/g)	*M*_r_ (emu/g)	*H*_c_ (Oe)	*M*_r_/*M*_25kOe_ (%)	(*BH*)_max_ (Cal.) (MG·Oe)
0.00	71.364	38.233	7035.1	53.6	1.65
0.05	71.862	38.427	7310.9	53.5	1.72
0.10	71.647	38.320	7580.4	53.5	1.84
0.15	72.837	38.688	7745.5	53.1	1.99
0.20	72.647	38.854	7880.4	53.5	2.01
0.25	71.401	38.567	7471.9	54.0	1.79
0.30	69.830	37.429	7415.9	53.6	1.67
0.40	66.886	35.288	7388.6	52.8	1.57
0.60	60.426	31.510	7178.0	52.1	1.13

From this viewpoint, the plate-like, Ca-substituted ferrite shows intriguing results. As a measure of crystallinity, the apparent full width at half maximum intensity (FWHM) of the (107) peak and the calculated crystallite size were determined from the XRD data, as shown in Fig. S6. The decrease in FWHM with increasing Ca^2+^ content resulted in an increasing crystallinity of the Ca-substituted Sr-ferrite particles, maintaining the level of *M* in spite of the foreign Ca introduction. The onset of a decline in *M* from *x* = 0.30 is attributed to the presence of byproducts, which in good agreement with the XRD data.

The Ca-substitution also induced a change in the microstructural characteristics, specifically from a spherical particle shape to a flat hexagonal plate, and this could have led to a strong decrease of *H*_c_. Nevertheless, up to *x* = 0.60, *H*_c_ remained at a high level, increasing up to 12% for *x* = 0.20 without a significant deterioration of *M*. Thus, the *H*_c_ tendency was not greatly influenced by the change in particle diameter and aspect ratio, implying that there must be another predominant factor having the greatest effect on *H*_c_.

To confirm the effect of Ca-substitution on the magnetocrystalline anisotropy, which can determine the highest achievable *H*_c_, we determined the first anisotropy constant through the law of approach to saturation (LAS) method.

### Causality between Ca-substitution and coercivity enhancement

The LAS theory is a popular method for determining the local crystalline anisotropy of magnetic materials, describing the empirical *H* dependency on *M*, in the form Eq. 3:

3 $$M = M_{s} \left\{ {1 - \left( {\frac{{\text{A}}}{H}} \right) - \left( {\frac{B}{{H^{2} }}} \right)} \right\} + \chi_{p} H$$

where *A*/*H* is the inhomogeneity of the materials, *χ*_p_*H* is the field-induced forced magnetization term, and *B*/*H*^2^ is a term associated with the magnetocrystalline anisotropy parameter^13^.

**Figure 4:**
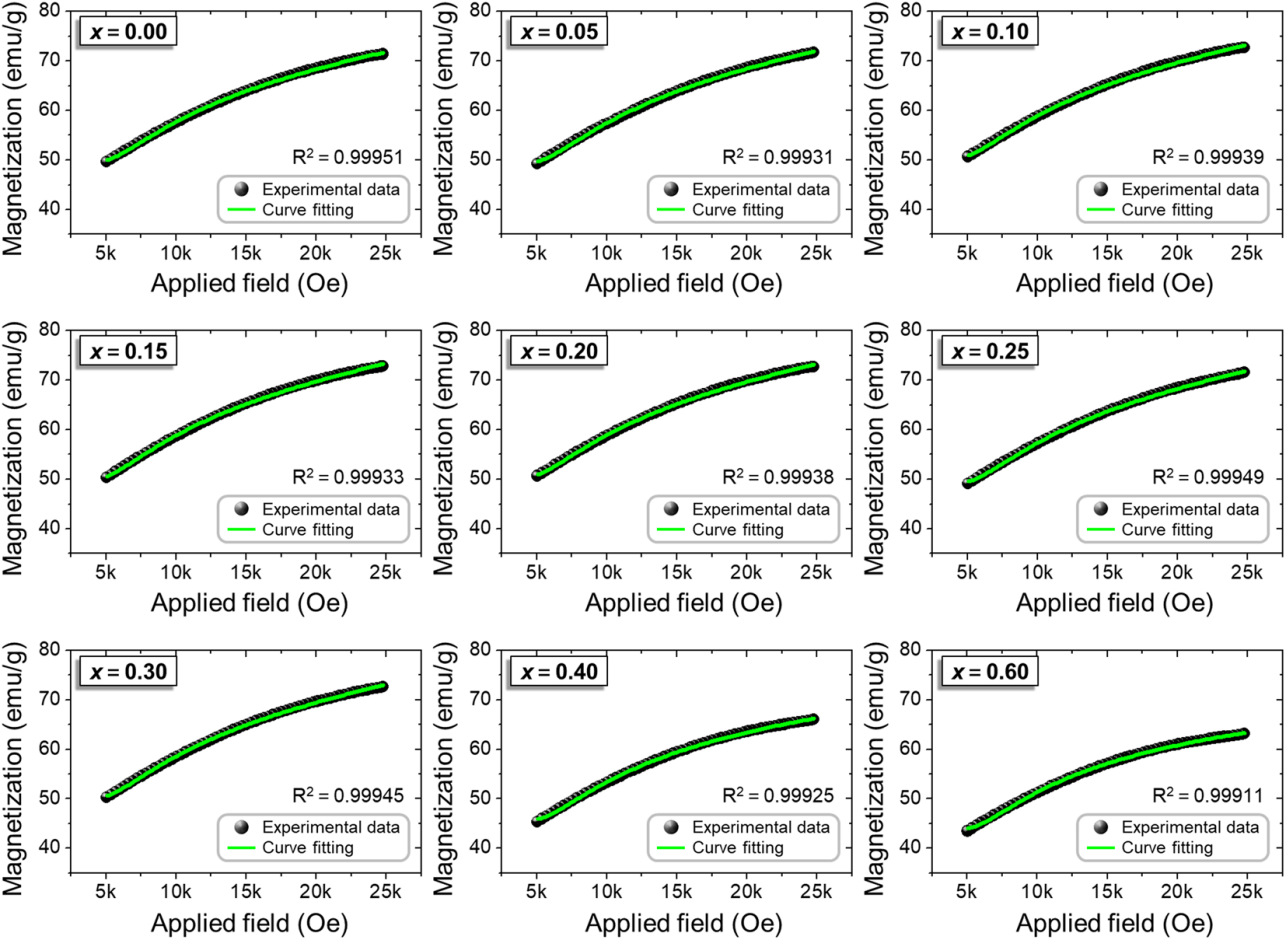
Fitting to the law of approach to saturation (LAS) and the corresponding values of the goodness of fit (*R*^2^) for Ca-substituted ferrite nanoparticles as a function of Ca-substitution contents (0.00 ≤ *x* ≤ 0.60).

Through Eq. 3, a typical curve fitting of experimental data with the output statistical parameter *R*^2^ (representing the goodness of the curve fit) is shown in Fig. 4. The results fit the curve with high reliability, with an *R*^2^ coefficient of determination values above 0.999. This indicates that all of the hexaferrite particles possess a good relationship between *M* and *H* and the *M* does not depend on any one specific term (see the data fitting to the LAS in different equation forms in Fig. S7 and Table S1).

Along with the *R*^2^, the fitted parameters also provide important information associated with the magnetic properties (Table 3): the drastic increase in the inhomogeneity parameter *A* for *x* > 0.30 can be understood, as the increase in structural defects and nonmagnetic ion inclusions resulted in a secondary phase formation^35^. For the hexagonal crystal structure, the anisotropy factor *B* can be expressed as:

4 $$B = \frac{{\left( {H_{A}^{2} } \right)}}{15} = \frac{{\left( {4K_{1}^{2} } \right)}}{{\left( {15M_{s}^{2} } \right)}}$$

**Table 3:** Values of *A*, *B*, *χ*_p_, *R*^2^, *H*_A_, and *K*_1_ of the M-type Sr_0.75-*x*_La_0.25_Ca_*x*_Fe_11.8_Co_0.2_O_19_ ferrite samples obtained from the fitting data (Fig. 4).

Ca^2+^ content (*x*)	*A* (× 10^3^)	*B* (× 10^6^)	*χ*_*p*_(× 10^–4^)	*R*^2^	*H*_A_ (Cal.) (kOe)	*K*_1_ (Cal.) (× kJ/m^3^)
0.00	2.7792	15.6282	3.4675	0.99951	15.311	289.55
0.05	2.9710	16.2676	3.6570	0.99931	15.621	297.48
0.10	2.9983	16.4826	3.8692	0.99939	15.724	298.54
0.15	3.0046	17.6702	3.9233	0.99933	16.280	314.24
0.20	2.9983	18.2826	3.8692	0.99938	16.560	318.81
0.25	3.0847	18.2278	3.5873	0.99949	16.535	312.87
0.30	3.0714	17.1724	3.8744	0.99945	16.049	309.07
0.40	3.6815	17.1256	1.7774	0.99925	16.028	284.09
0.60	3.9001	16.7892	8.1328	0.99911	15.869	254.12

where *H*_A_ is the anisotropy field, and *K*_1_ is the magnetocrystalline anisotropy constant. On the basis of the fitting results, *K*_1_ and *H*_A_ were calculated by using Eqs. 4 and 5:

5 $$H_{A} = \frac{{2K_{1} }}{{M_{S} }}$$

Up to *x* = 0.20, the *K*_1_ and *H*_A_ first gradually increased with increasing Ca content (*x*): from 289.55 to 318.81 kJ/m^3^, and from 15.311 to 16.560 kOe, respectively, which was similar to the behavior of *H*_c_ (i.e., from 7035.1 to 7880.4 Oe) in Sr_0.75−*x*_La_0.25_Ca_*x*_Fe_11.8_Co_0.2_O_19_. While *M* was steadily maintained (71–72 emu/g), the increases in *K*_1_ and *H*_A_ can be attributed to both the predominant lateral growth in the hexagonal unit cells, and plate-stacking of the ferrite particles along the easy-axial orientation. For *x* > 0.20, the *K*_1_ and *H*_A_ values decreased, possibly due to the deteriorated *M* and purity of the ferrite particles and a decreased amount of stacked particles. In terms of the anisotropy factors, *H*_c_ can be expressed as follows^36^:

6 $$H_{c} = \alpha \left\{ {\left( {\frac{{2K_{1} }}{{M_{s} }}} \right) - \left( {N_{d} M_{s} } \right)} \right\} = \alpha \left( {H_{A} - H_{d} } \right)$$

where *α* is the shape constant and *N*_d_ and *H*_d_ are the terms related to the demagnetizing coefficient.

**Figure 5:**
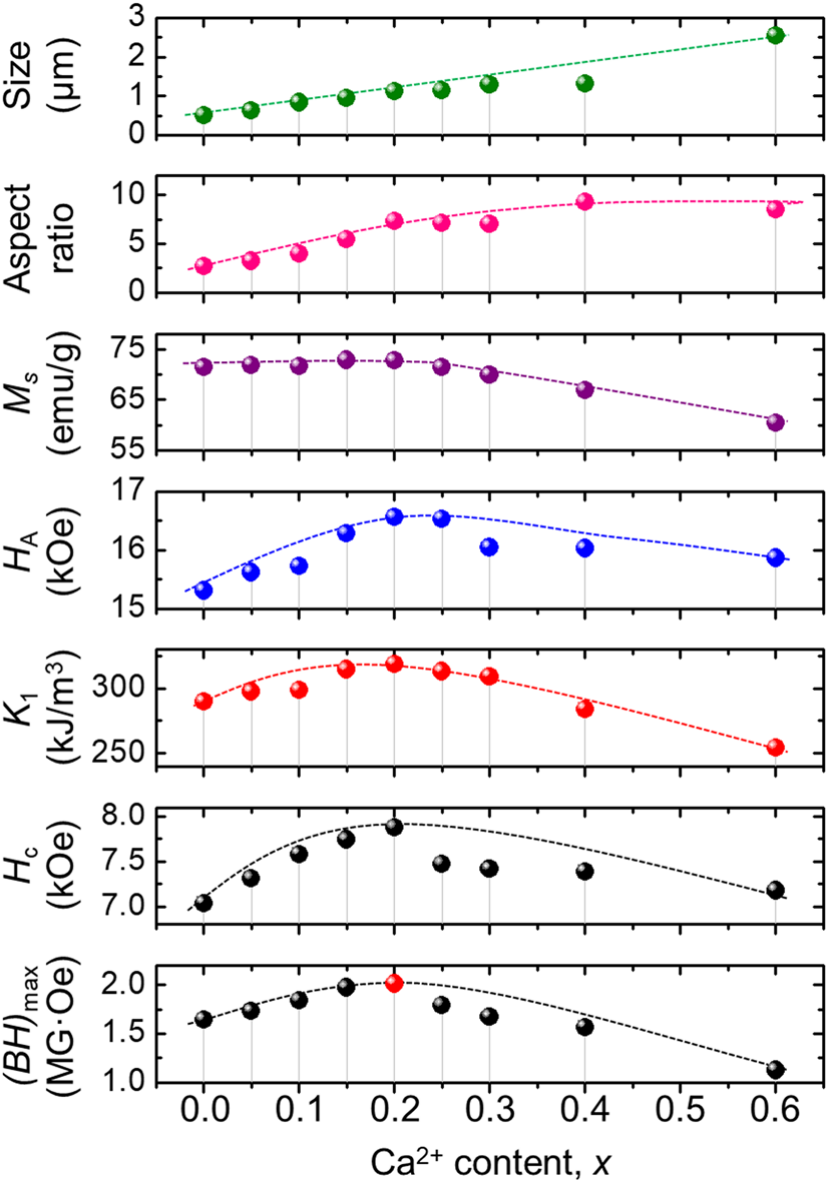
Dependence of the particle diameter, aspect ratio, and magnetic properties on Ca-substitution content (0.00 ≤ *x* ≤ 0.60) in Sr_0.75−*x*_La_0.25_Ca_*x*_Fe_11.8_Co_0.2_O_19_.

Since *H*_c_ is proportional to *K*_1_/*M*_s_ and *H*_A_ on the basis of Eq. 6, *H*_c_ improved remarkably, up to 7880.4 Oe (about > 800 Oe) alongside *K*_1_, in the optimized Sr-lean composition. Consequently, the combination of high *M* and large *H*_c_ results in a calculated maximum energy product ((*BH*)_max_) enhancement of 2.01 MG·Oe, over 120% of the value of Sr-ferrites with no Ca-substitution (1.65 MG·Oe). Figure 5 summarizes this empirical dependency.

## Conclusion

In summary, Ca-substituted Sr-hexaferrite plates possessing partial La^3+^–Co^2+^-substituents (Sr_0.75−*x*_La_0.25_Ca_*x*_Fe_11.8_Co_0.2_O_19_; 0.00 ≤ *x* ≤ 0.60) were successfully synthesized via the ultrasonic spray pyrolysis process. Interestingly, simultaneous enhancements in the intrinsic coercivity (*H*_c_), without sacrificing magnetization (*M*), were achieved by Ca-substitution: 20 at.% of Ca-substitution for Sr exhibited the largest *H*_c_ of 7880.4 Oe with a *M*_s_ of about 72.6 emu/g and thereby an enhancement in a maximum energy product ((*BH*)_max_) of 2.01 MG·Oe, compared to Sr-hexaferrite with *x* = 0.00 (*H*_c_ of ~ 7035.1 Oe; *M*_s_ of ~ 71.4 emu/g; (*BH*)_max_ of 1.65 MG·Oe). Through microstructural and magnetic studies, we found that the additional Ca-substitution led to dramatic changes in the anisotropic characteristics of the Ca-substituted Sr-ferrite, specifically, more plate-like-shaped particles ascribed to pronounced lateral growth, and a strong increase in its magnetocrystalline anisotropy, *K*_1_, far beyond the optimized La–Co-substituted Sr-ferrite. As a result, the optimal composition contained earth-abundant Ca, to the benefit of both enhanced magnetic performance and lower composition costs as compared to the current commercial ferrite magnets.

We expect that this new Sr-lean composition, possessing enhanced magnetic properties, will find applications far beyond the limitations of traditional Sr-ferrite-based magnetic materials, and we envision that it can be widely used in new types of affordable magnets.

## Supplementary information

Supplementary file1
