# Supplementary material for: Anisotropic characteristics and improved magnetic performance of Ca–La–Co-substituted strontium hexaferrite nanomagnets
Source: Sci Rep. 2020 Sep 28;10:15929. doi: 10.1038/s41598-020-72608-0 (PMC7522080; doi:10.1038/s41598-020-72608-0)
Supplement: Supplementary file 1 — Supplementary file1 [file 41598_2020_72608_MOESM1_ESM.pdf]

Supplementary Information for:

Anisotropic Characteristics and Improved Magnetic Performance of Ca-La-Co-substituted Strontium Hexaferrite Nanomagnets

Jimin Lee[1], Eun Jae Lee[1], Tae-Yeon Hwang[2], Jongryoul Kim[1], and Yong-Ho Choa[1,*]

[1]Department of Materials Science and Chemical Engineering, Hanyang University, 55, Hanyangdaehak-ro, Sangnok-gu, Ansan-si, Gyeonggi-do 15588, Korea
[2] Center for Quantum Information, Korea Institute of Science and Technology (KIST), 5, Hwarang-ro 14-gil, Seongbuk-gu, Seoul 02792, Korea

*Corresponding author:
choa15@hanyang.ac.kr, Tel: +82-31-400-5650, Fax: +82-(31)-418-6490 (Yong-Ho Choa).

FIGURE

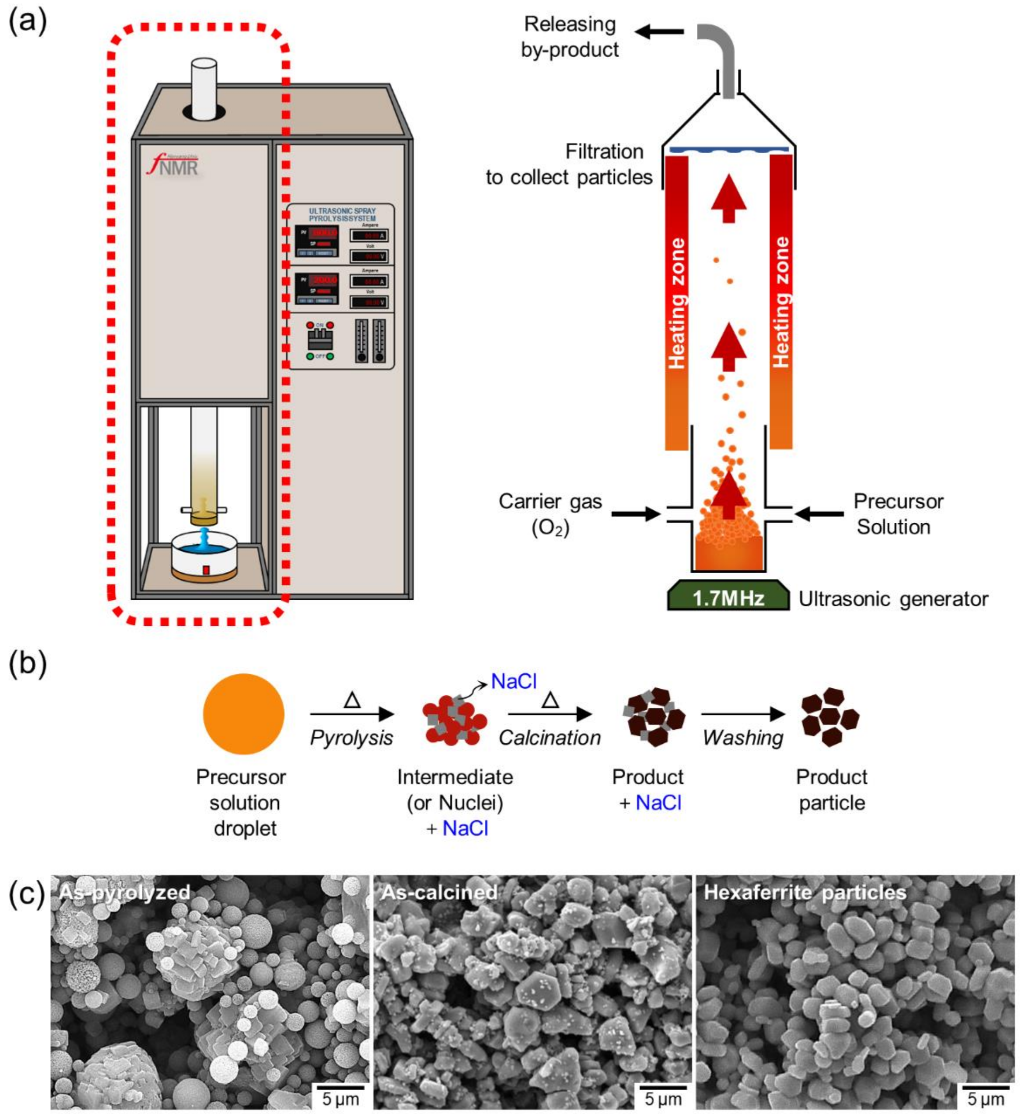

Figure S1 (a) Schematic of a laboratory-scale USP setup; **(b, c)** phase and morphology of the particles obtained after each step.

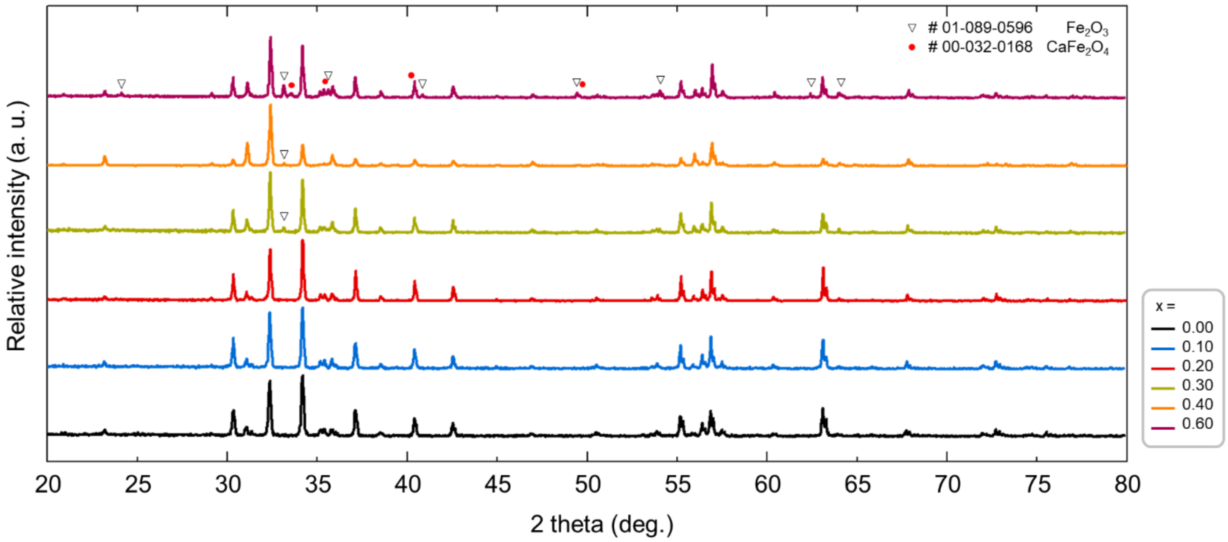

Figure S2 Normalized X-ray diffraction patterns of the series $Sr_{0.75-x}La_{0.25}Ca_xFe_{11.8}Co_{0.2}O_{19}$ (x = 0.00, 0.10, 0.20, 0.30, 0.40 and 0.60).

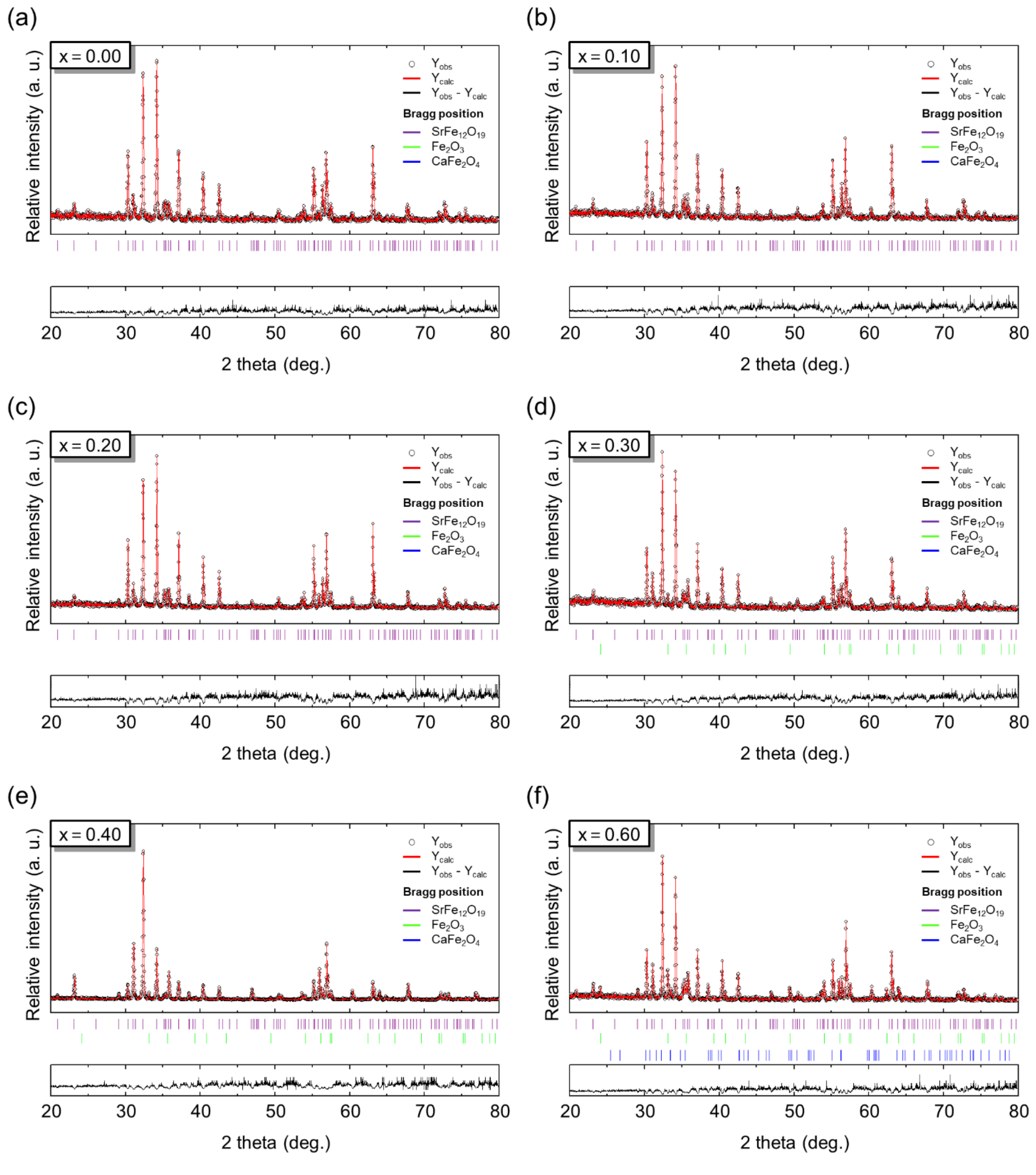

Figure S3 Rietveld analysis for X-ray diffraction pattern of $Sr_{0.75\text{-}x}La_{0.25}Ca_xFe_{11.8}Co_{0.2}O_{19}$ (x = 0.00, 0.10, 0.20, 0.30, 0.40 and 0.60).

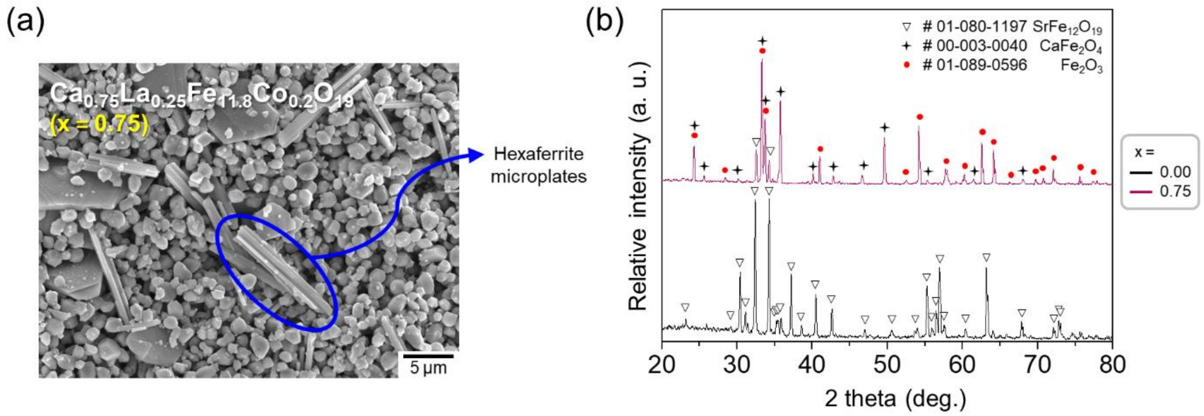

Figure S4 (a) FE-SEM micrograph and **(b)** corresponding normalized X-ray diffraction patterns of the $Ca_x La_{0.25}Fe_{11.8}Co_{0.2}O_{19}$ (x = 0.75; No Sr source) particles.

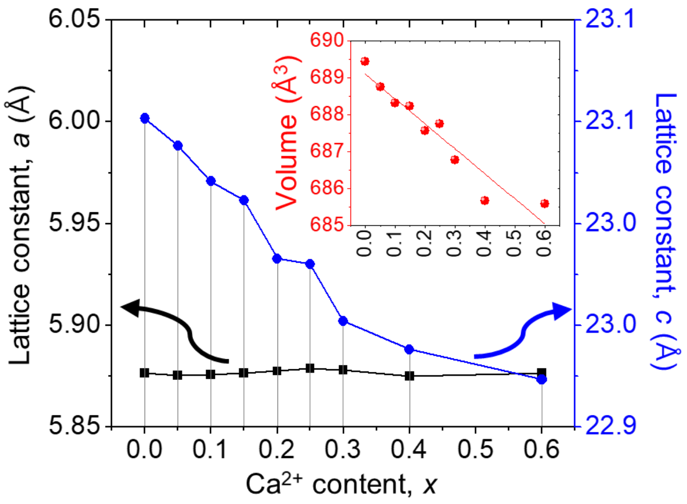

Figure S5 Lattice parameters *a*, *c* and lattice volume as a function of *x* in $Sr_{0.75-x}La_{0.25}Ca_xFe_{11.8}Co_{0.2}O_{19}$ hexaferrites.

For the series of $Sr_{0.75-x}La_{0.25}Ca_xFe_{11.8}Co_{0.2}O_{19}$ samples with Ca content (*x*) from 0.00 to 0.60, the lattice constants *a* and *c* were calculated from the d_{hkl} values corresponding to the (107) and (114) peaks.

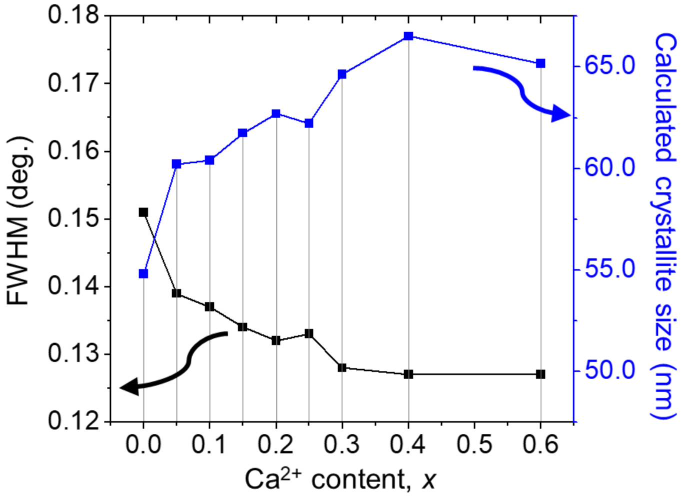

Figure S6 Dependence of the full width at half maximum (FWHM) value and calculated crystallite size upon Ca concentration.

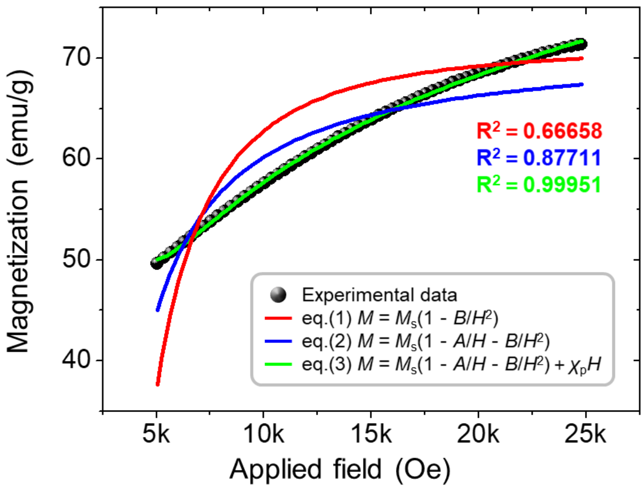

Figure S7 A typical curve fitting of experimental data of $Sr_{0.75-x}La_{0.25}Ca_xFe_{11.8}Co_{0.2}O_{19}$ (x = 0.00) particles using Eqs. (1) - (3).

Table S1 Values of A, B, χ_p, and R^2 of M-type $Sr_{0.75-x}La_{0.25}Ca_xFe_{11.8}Co_{0.2}O_{19}$ (x = 0.00) ferrites samples obtained from **Fig. S6**.

Equation	A	B	χ_p	R^2
(1)	-	1.1985×10^7	-	0.66658
(2)	1.2638×10^3	3.0035×10^6	-	0.87711
(3)	2.7792×10^3	15.6282×10^6	3.4675×10^{-4}	0.99951

It can be seen clearly that for the Sr-ferrites, the best fit resulted from Eq. (1). This reveals the collective dependency of magnetization on the A/H, B/H^2, and $\chi_p H$ terms, and not merely on the B/H^2 term.
